# From the shadows: A qualitative study of fathers' perspectives and experiences of their partner's gestational diabetes mellitus and its implications

**DOI:** 10.1111/dme.15488

**Published:** 2024-12-06

**Authors:** Madeleine Benton, Nabiha Waheed, Khalida Ismail

**Affiliations:** ^1^ Department of Psychological Medicine King's College London London UK

**Keywords:** fathers, gestational diabetes mellitus, partners, perinatal, type 2 diabetes

## Abstract

**Aims:**

Gestational diabetes mellitus (GDM) affects an increasing number of women each year. Research involving partners of women with GDM, such as fathers is limited, however, understanding their perspectives and involvement in GDM management could enhance health outcomes for both women and their families. This study aims to explore the impact and experiences of GDM on fathers in the perinatal period.

**Methods:**

Qualitative, individual, semi‐structured interviews were conducted with individuals whose partners either currently had GDM or had been diagnosed with GDM within the past 3 years. All participants identified as biological fathers. Data were transcribed and analysed using reflexive thematic analysis.

**Results:**

Nine fathers participated in the study. Analysis resulted in four themes that explored fathers' experiences of GDM during the perinatal period: ‘navigating a GDM diagnosis’, ‘partnering in management and health’, ‘finding a place within the GDM journey’ and ‘the lasting impact of GDM’.

**Conclusion:**

Fathers encountered wide‐ranging impacts of their partners' GDM. Fathers provided both practical and emotional support to their partners. Engaging fathers in discussions at diagnosis and in management could facilitate the maintenance of positive health behaviour changes postpartum, potentially reducing the risk of type 2 diabetes for both parents.

**What's new**

**What is already known?**

- Social support, including that from partners, is important during pregnancy.
- Women with previous gestational diabetes mellitus (GDM) are at increased risk of developing type 2 diabetes (T2D), as are their children and partners.

**What this study has found?**

- Fathers reported providing emotional and practical support to their partners during a GDM pregnancy.
- Fathers encountered challenges in implementing and maintaining diet and physical activity changes alongside their partners.
- Few fathers were aware of their potential increased risk of developing T2D.

**What are the implications of the study?**

- Engaging fathers in diagnostic and management discussions alongside their partners could enhance their awareness of T2D risk factors and facilitate the adoption of health‐promoting behaviour modification.

## 1 BACKGROUND

Gestational diabetes mellitus (GDM), characterised by hyperglycaemia during pregnancy, affects around 10% of pregnancies each year in the United Kingdom (UK) with its prevalence increasing.^1^ The potential maternal, obstetric and neonatal adverse outcomes of GDM are well established and can include preeclampsia, macrosomia (a neonate weighing over 4 kg), caesarean section, shoulder dystocia, instrumental birth, and birth injury.^2^

GDM management centres on optimising glycaemic targets to improve pregnancy outcomes. Diet and physical activity modification is recommended as the primary therapeutic strategy for women with GDM.^1^ If management through physical activity and diet modification alone is not sufficient to achieve glycaemic targets, insulin therapy or metformin may be indicated.^1^ As part of GDM management, women are also required to monitor their blood glucose multiple times a day and attend additional medical appointments. Consequently, GDM pregnancies can become highly medicalised. Furthermore, increasing evidence highlights the negative psychosocial effects of GDM on women,^3^ including a 50% increased risk of postnatal depression.^4^

Although GDM typically resolves after birth, it carries long‐term adverse risks for both mother and baby. The mother is at increased risk of GDM recurrence in subsequent pregnancies and up to a 10‐fold increased risk of developing type 2 diabetes (T2D) later in life.^5^ Infants are at increased risk of early‐onset obesity, T2D and neurodevelopmental disorders.^6^, ^7^ Further, research suggests a potential link between maternal GDM and an elevated risk of future T2D in fathers. Notably, a 33% rise in T2D has been observed among fathers whose partners had GDM.^8^ This could be attributed to the shared social and cultural environment of both parents, which can shape health behaviours.^9^

Research has consistently demonstrated the importance of social support in pregnancy.^9^ Recent research has highlighted the importance of partner support, for women with GDM, in improving mental health outcomes and encouraging positive behaviour change.^10^, ^11^ A systematic literature review on the needs and experiences of women with GDM reported that women with supportive partners demonstrated a higher likelihood of engaging in regular exercise and effectively monitoring their blood glucose levels.^12^ This support could extend beyond the antenatal period, to assist continued health behaviours in the postnatal period to reduce the risk of developing T2D later in life. Social exchange theories, such as interdependence theory, propose that partner support can facilitate behaviour change and help maintain each other's healthy behaviours.^13^ In the context of the present study, interdependence theory suggests that partners may also adopt positive behaviour change by mirroring the behaviours encouraged in women with GDM. Previous research has found that positive health behaviour changes, such as increases in physical activity, in one partner tend to be reflected in the other partner.^14^ This suggests that women's management of GDM can positively influence their partner's, potentially also reducing their risk of developing T2D. Further, such research suggests that including partners in GDM management programmes may translate to better outcomes for women with GDM and a reduced risk of T2D in the future for both parents.^15^

Research on the impact of a GDM diagnosis on partners is limited. However, understanding their perspectives and involvement in GDM management could enhance health outcomes for both mothers and families. This study aims to investigate the impact and experiences of GDM on partners, including their knowledge of the condition.

## 2 METHODS

### 2.1 Design

A qualitative study design using semi‐structured individual interviews was employed. This provided a standardised structure while allowing for flexibility in exploring topics of interest in more depth.^16^ COREQ (COnsolidated criteria for REporting Qualitative research) reporting guidelines were followed (see appendices for COREQ Checklist).

### 2.2 Participants

Participants were recruited through purposeful sampling with the intention of recruiting a diverse group of fathers, including those with partners at different gestational stages, with varying numbers of children, and from different ethnic groups.^17^ Eligibility criteria included (i) partners of women currently diagnosed with GDM or with a GDM diagnosis in the past 3 years, (ii) at least 18 years of age and (iii) able to read and speak in fluent English. Interested participants e‐mailed the research team and were sent detailed information about the study. Interview times were scheduled with consenting participants. Recruitment took place online between November 2022 and April 2023 through advertisements distributed on social media platforms and shared within pregnancy support groups.

### 2.3 Data collection

An interview schedule was developed a priori with input from an expert advisory group including seven women with current or past experiences of GDM, of varied ethnicities, and number of GDM and pregnancy experiences. The interview schedule was then piloted with one father to gauge flow of interview questioning. This pilot interview was included in the final data set. Questions were adapted as interviews progressed, depending on the themes identified, and the content of interviews. The final interview guide included topic areas such as fathers' understanding of GDM, experiences of GDM diagnosis and management, their role in the GDM experience in the antenatal and postnatal period, and family health behaviour change. The first author (MB) is an experienced qualitative researcher in women's health and conducted the interviews. Interviews were undertaken virtually using video‐conferencing software and recorded. Interviews ranged in length between 26 and 43 min (mean time = 31 min). Audio recordings of the interviews were transcribed verbatim and anonymised.

Demographic data were collected at the beginning of the interview to aid description of the sample, including age, parity, antenatal or postnatal status and ethnicity (Table 1).

**TABLE 1:** Demographic characteristics of participants.

Participant number	Age (years)	Ethnicity	Partner in the antenatal or postnatal period	Parity
1	32	White British	Postnatal	1
2	40	White British	Postnatal	2
3	33	White British	Postnatal	1
4	38	White British	Postnatal	2
5	40	White British	Postnatal	1
6	35	White Other	Postnatal	1
7	33	White Other	Antenatal	2
8	34	White British	Postnatal	1
9	34	White British	Postnatal	2

### 2.4 Analysis

Data were analysed using reflexive thematic analysis, a method offering flexibility and variability in theoretical and analytic scope. It allowed for identification, analysis and reporting of meaningful patterns and themes from the data.^18^ The present analysis was seated in a critical realist framework. This approach meant we treated participants' experiences as real and true to them, yet inextricably mediated, shaped and conditioned (socially), at the intersections of cultural context and by different aspects of the individual.^19^ An empathetic and objective outsider position within the data was adopted, as none of the researchers involved were fathers, partners of someone with GDM or had experienced GDM themselves. Analysis followed six steps outlined by Braun and Clark.^18^ Firstly, data were familiarised by two researchers (MB, NW) who read and re‐read transcripts and reviewed recordings to become intimately familiar with the data. Secondly, coding of the whole data set was undertaken which included shorthand descriptions and interpretive labels for pieces of information within the data set that were relevant to the research question. Next, coded data were reviewed and analysed, MB and NW discussed how different codes could be combined according to shared meanings which formed themes or sub‐themes, which were then further scrutinised. Theme names were then revised and quotes to illustrate the themes were selected.

### 2.5 Ethics

Ethical approval was granted by the King's College London Research Ethics Committee (LRS/DP‐21/22‐33, 198). To ensure anonymity of participants, all identifiable information was removed in transcription.

## 3 RESULTS

Nine partners of women with current or past GDM diagnosis were interviewed (mean age = 35 years old). All partners identified as fathers and are therefore referred to as father's hereafter.

Analysis resulted in four overall themes, derived from fathers' experiences of GDM in the perinatal period: (1) navigating a GDM diagnosis, (2) partnering in management and health, (3) finding a place within the GDM journey and (4) the lasting impact of GDM.

### 3.1 Theme 1: Navigating a GDM diagnosis

#### 3.1.1 Knowledge and beliefs about GDM

Participants' understanding of GDM, its causes and implications for mother and baby varied. Most participants had minimal knowledge of the condition prior to diagnosis, and for many, it was their first‐time hearing about the condition at diagnosis.

Up until my wife developed it… I hadn't even heard of gestational diabetes… so my understanding [now] is it's to do with blood sugar levels. And they need to be… controlled with things like metformin… in order to bring them down to more controllable level, because otherwise there can be impacts on the baby. They can grow too quickly…. So it's generally not a good thing… I was told if it's noticed early enough and managed correctly, then it's not the end of the world normally. (P3)

I didn't [have] any knowledge of GDM, it was a bit scary… trying to work out what causes it, or is it something that we've done? Or [wife's name] done that could cause it or lifestyle… but I think initially it was just a fear of what does this mean. (P5)

Several participants discussed recognising that GDM is more prevalent than they had originally thought.

… it turns out it [GDM] was more common when you speak to folk, but nobody seems to talk about it… but once you speak to folk they'll say ‘oh my sister had that' or 'this person had that’ and it is more common, I think, than what I expected, because I'd never heard of it. (P5)

#### 3.1.2 Reactions at diagnosis

Initial reactions to a partner's diagnosis included worry, surprise and shock. Fathers expressed empathy towards their partners as they were not the ones receiving the direct diagnosis.

I wasn't very happy… I felt a bit hollow, it seems daft in hindsight, but I heard diabetes and I heard like cancer. I heard death sentence. It sounded like lots of people with diabetes do die early. It was quite tough to hear that my wife… had diabetes of any kind. (P4)

The extent and causes of concern differed among fathers.

Well, I think rather selfishly, my worry was for the baby rather than my wife… It's naturally selfish, but I think the more I thought about it, the more it was it sort of evenly spread. (P3)

Fathers described stigma surrounding the condition, particularly related to its causes. Stigma was described as being experienced by oneself, close family and broader society.

There's a presumption that the people who suffer from this [GDM], are, I don't know, less well educated, poorer, overweight. (P8)

### 3.2 Theme 2: Partnering in management and health

#### 3.2.1 Challenges in interactions with healthcare professionals

Fathers' interactions with healthcare professionals (HCPs) were mixed, with frustration expressed related to information provision around management as well as related to birth choices.

Actually, the information is kind of pathetic… It felt a little…‘oh, you know, try not to eat cakes’. Yeah, fantastic, thanks for that advice, we had never thought about that. Is there any other advice that we can be given? (P8)

Feelings of joint participation were also discussed as a result of the perceived inadequate advice from HCPs regarding management. Couples decided to resort to online resources to gain further information that they felt was relatable and reassuring.

Some of the gestational diabetes sites that they have now and forum groups and other people that have got shared experiences, I think it's invaluable… I know the second time round that's where my wife went straight away. (P2)

#### 3.2.2 Reassurance through increased contact with healthcare professionals

Some fathers described positive elements of GDM including the provision of increased monitoring and scans where they could see their baby.

If anything, we probably got more touch points over the pregnancy… we were seen every four weeks… and we went to the hospital for extra scans. (P4)

Sounds a wee bit selfish, but that at least it gave us more chance to ensure the pregnancy was going the way we hoped it to go, so it wasn't always the worst thing. Knowing that you're going getting another scan done. (P5)

#### 3.2.3 Shared frustration with the impact on pregnancy and labour

Fathers described a shared sense of disappointment with their partners, as they highlighted that GDM impacted the overall enjoyment of the pregnancy experience. They described that the focus on monitoring, management and dietary changes disrupted what they perceived should be a normative pregnancy experience.

The GDM… just exacerbated an already bad situation… there was, you know, some days where she'd be a bit down in the dumps and then have to take her insulin and she'd be even more down in the dumps about that. (P4)

Being so fixated on your numbers, your diet, your management is taking away from the experience of just being pregnant… All the conversations became about… how we were managing this. (P7)

Several fathers highlighted the negative impact GDM had on labour and birth choices and frustration when both them and their partner felt the condition was well managed, yet felt options around birth were taken away.

[GDM] just destroyed them [birth plans]… when it came to the actual birth… there was no issues with the baby's size, but they decided anyway, no, because it's this, we will bring you in for an induction… it was quite a horrible experience for [wife's name] all in all to be honest, cause they'd try to induce her, the baby wasn't ready, her body wasn't ready for it. And then eventually… it was like, we'll, give you a [Caesarean] section… it wasn't a nice ending. (P5)

### 3.3 Theme 3: Finding a place within the GDM journey

#### 3.3.1 Offering practical support

Fathers discussed recognising their limited direct influence on the pregnancy, which led them to seek alternative ways to provide support. Recognising the need for a varied support role, fathers actively engaged in practical aspects, often assuming increased responsibilities, including meal preparation and also changing their own diet to support women with their often very restricted GDM diet.

As the father, you have very little input, you know, so obviously [wife's name] is carrying this child and everything with the child's health. I can do certain things. I can run about and get things. But [wife's name] doing everything really so, watching or checking my diet, it's not really a big ask, is it for that length of time. (P5)

#### 3.3.2 Providing emotional support

Although some fathers discussed being able to provide practical support, many perceived their primary role as emotional support providers.

There was definitely an emotional role in terms of trying to help support my wife… in taking every day as it comes without beating yourself up for you know having the perfect diet and exercise plan. (P3)

I was also putting myself into my wife's shoes and trying to think what she'd be feeling and working out what I could do to comfort her… just trying to understand what the right things to say to her, and giving her whatever support, any unqualified support, that I could was at the forefront of my mind. (P4)

#### 3.3.3 Challenges faced during the journey

The main difficulties reported by fathers were related to managing dietary changes. Many fathers who took on roles to assist with meal preparation reported feeling overwhelmed and lacking confidence in knowing how or what needed to change.

I wasn't particularly capable of doing you know, a perfect diet… of cooking and preparing a perfect diet for someone with GDM. (P3)

Fathers expressed difficulty in attaining the ‘perfect lifestyle’. This included challenges in adapting their own diets as they felt it was a choice, not a necessity. This was reflected in fathers' inconsistency in adhering to nutritional advice, with some reporting a lack of transparency when communicating this with their partners.

I tried to… There was definitely also some like secret eating. If I felt that actually, you know I would like some more to eat but didn't want to kind of be, rubbing it in basically. (P3)

### 3.4 Theme 4: Lasting impact of GDM

#### 3.4.1 Increased awareness of GDM and its consequences on women

Most fathers reflected on their increased awareness of GDM and, for some, the increased risk of type 2 diabetes more generally. There was little awareness of the impact of GDM on fathers themselves.

If my wife develops diabetes type 2, I think that would be a shock to the system… and require a significant re‐evaluation of how we live our lives. (P1)

It [GDM] was an eye opener for me as well… she [partner] had it, but I could easily have the type 2 as well…we don't have a family history, but it doesn't necessarily mean that I'm immune to it… So it definitely put it to the back of my mind. I think just probably a bit more nudges needed to… get to the front of my mind as well and make more active lifestyle changes. (P6)

Other concerns fathers held were regarding the lasting emotional impact of GDM on women, with some noticing considerable health anxiety in women during the postnatal period.

I knew that this [GDM] was going to have quite the effect on her and still does… the end result was far more traumatic than the first‐time round… I think that's why my wife is still mentally scarred by it. (P2)

#### 3.4.2 Maintaining postnatal behaviour change

The maintenance of behaviour changes including to nutrition and exercise in the postnatal period varied considerably. Many fathers described intentions of maintaining change, but barriers included child caring duties and juggling day‐to‐day living and activities including returning to work. Many often reflected on increased consideration for their diets and understanding of different foods, but often did not engage in lasting dietary changes.

It's very easy in the course of day‐to‐day, of family life to kind of slip into, well everyone's eating fish fingers, basically, you know the easy stuff… I wasn't particularly capable of… preparing a perfect diet for someone with GDM, whilst juggling a 3‐year old's diet. (P3)

Nonetheless, many participants discussed their children as a motivator for change to behaviours and recognised that they were able to model healthy eating habits.

I try and make a conscious effort to have more vegetables to just show the right way to the children. (P2)

#### 3.4.3 Planning for future pregnancies

Interestingly, most fathers, experienced reduced concern about future pregnancies due to the sense of control and agency gained from managing GDM, which encouraged reflection and a re‐evaluation of their expectations during pregnancy.

If we were planning on having a third [child], the fact that my wife could contract gestational diabetes wouldn't be a factor in the choice that we made. (P4)

Whereas, some fathers described the negative impact GDM had on decisions related to future reproductive health.

We probably won't try and have another one because I couldn't bear, and she couldn't bear to go through that amount of injections. (P9)

I think it [GDM] changed my view… it's a consideration, if we were to suggest having a second child. It [the pregnancy] would be diet controlled, which did become harder the further we went on and, especially for [wife's name], cause she was dealing with it, but it would be a consideration also before having a second [child]. (P5)

## 4 DISCUSSION

This study provides a detailed exploration of the impact of GDM on fathers during the perinatal period. Four themes were generated encompassing fathers' initial perceptions of GDM, the mutual impacts of GDM shared between couples, the various roles undertaken by fathers and the perceived lasting effects of GDM.

Many fathers were unaware of GDM before their partner's diagnosis, despite its growing prevalence as a pregnancy complication. This is in line with recent literature suggesting that fathers' lack of knowledge of the condition may be due to GDM education, often purely targeted at women.^11^ Fathers felt concerned and distressed for the mother and baby at diagnosis and during management, alongside disappointment with the impact of the condition on the pregnancy experience. Despite previous research identifying social isolation as a significant consequence of GDM for women, fathers did not report feeling isolated, indicating an inherent difference in how experiences are perceived by those diagnosed with GDM and their partners.^20^ Fathers highlighted they recognised stigma surrounding GDM at interpersonal, intrapersonal and societal levels and the negative impact this could have on women. Many fathers also recognised their own stigma surrounding the condition which was challenged with their partner's diagnosis. The adverse impacts of stigma on various health outcomes have been well recognised and recent research has underscored the multifaceted nature of stigma surrounding GDM and emphasised its convergence with weight stigma during pregnancy.^21^, ^22^ Several short‐term consequences of GDM‐specific stigma have been identified including self‐blame, guilt, shame, anxiety, isolation and impact on pregnancy decisions, which can indicate a potential high risk of long‐term consequences for women who experience stigma around the condition.^23^

Fathers discussed providing an emotional support role including a space for their partner to express frustration with managing GDM. This is in line with previous findings that have identified the importance of emotional support from fathers in improving the psychological burden experienced by women with GDM.^12^ Fathers perceived playing a practical support role through meal preparation, assisting in checking their partner's blood glucose levels, and taking increased responsibility of household tasks. A key aspect of support for some fathers involved adjusting their own health behaviours alongside their partner. However, this decision appeared to be influenced by their level of motivation and the practicality of the changes. Overall, fathers acknowledged the impact of GDM management on women and believed that providing both emotional and practical support may reduce the individual burden experienced by women.

Fathers who modified their diet alongside their partners reported a shared acknowledgement of the challenges involved with such change. Interestingly, previous research has demonstrated women with GDM report that it is more difficult to adhere to strict dietary management when their partners are not following the same diet.^24^ Increasing awareness among fathers about the mutual impact of their actions on their partners' health might encourage them to consider adjustments to their own health behaviours, supporting the theory of interdependence. This potential shift could contribute to better management adherence strategies, improved blood glucose management for women with GDM and sustained health behaviour change, consequently reducing the risk of developing T2D for the couple. This underscores the importance of a tailored approach to diabetes preventions including the entire family unit in postnatal education and support around health behaviours.

Participants identified a need for improved delivery of information from healthcare professionals, as most fathers expressed their reliance on the Internet for health‐related information. Although there are advantages to using the internet to source information and as a means for peer‐support, this should not replace traditional sources but rather supplement them.^25^ Fathers also revealed feeling abandoned by the healthcare system, as a result of the lack of follow‐up which has also been described from women's perspectives in literature.^26^ The postnatal period and time of follow‐up could be a vital time to discuss the maintenance of positive health behaviour changes^27^ and assist in reducing fathers' and their partner's future risks of developing T2D. Importantly, many participants expressed concerns primarily regarding the impact of GDM on women and their risk of developing T2D in later life, which contrasts with findings of a recent study which reported a general lack of awareness of the increased risk of T2D after GDM.^20^ However, although many fathers were aware of women's increased risk of T2D after GDM, most were not aware of the potential increased risk for themselves, which is in line with previous research.^8^

### 4.1 Limitations

We experienced considerable difficulty in recruiting fathers for this study despite utilising a range of recruitment methods. Challenges in recruiting men to this area of research have been acknowledged by others in the field, in addition to men often being overlooked in perinatal health.^28^ An additional important limitation is the lack of ethnic diversity within our sample and the exclusion of fathers who do not speak English, groups who may have provided experiences different to the English‐speaking partners included.^29^ We also recruited only one father who was currently in the antenatal period, with the remainder having to recall experiences up to 3 years earlier. However, this also may have provided participants time to cogitate and deeply reflect on experiences which may have made it easier for fathers to articulate their experiences.

### 4.2 Implications and future recommendations

The findings emphasise the important role fathers play in the GDM experience, in addition to the impact GDM can have on their own experience of the pregnancy. It highlights the significance of including fathers within aspects of GDM care, including diagnosis and management, to enhance adherence to behaviour change including diet and physical activity in both women with GDM and their partners. Previous findings have supported targeting couples rather than individual women, indicating the importance of including fathers in behaviour change interventions.^30^ Increasing partner engagement in care, including participation in consultations, and promotion of behaviour changes, could enhance long‐term outcomes in men as well as women, reducing their risk of T2D and improving overall health.

### 4.3 Conclusion

This study emphasises the wide‐ranging impacts of a partner's GDM on fathers, highlighting the practical and emotional support they provide during this time. Findings suggest that involving fathers in discussions throughout their partner's GDM journey could facilitate the maintenance of health behaviours after GDM. In the long term, this may help to decrease the risk of T2D for women with GDM and their partners.

## FUNDING INFORMATION

No funding was received for this study.

## CONFLICT OF INTEREST STATEMENT

The authors declare no conflict of interest.

## Supporting information


**Data S1.** Supporting Information.

## Data Availability

Research data are not shared.

## References

[1] NICE . Diabetes in Pregnancy: Management from Preconception to the Postnatal Period . 2020. 32212588

[2] Jovanovic L , Pettitt DJ . Gestational diabetes mellitus. JAMA. 2001;286(20):2516. doi:10.1001/jama.286.20.2516 11722247

[3] Benton M , Silverio SA , Ismail K . “It feels like medically promoted disordered eating”: the psychosocial impact of gestational diabetes mellitus in the perinatal period. PLoS One. 2023;18(7):e0288395. doi:10.1371/journal.pone.0288395 37478148 PMC10361484

[4] Wilson CA , Newham J , Rankin J , et al. Is there an increased risk of perinatal mental disorder in women with gestational diabetes? A systematic review and meta‐analysis. Diabet Med. 2020;37(4):602‐622. doi:10.1111/dme.14170 31693201 PMC7154542

[5] Vounzoulaki E , Khunti K , Abner SC , Tan BK , Davies MJ , Gillies CL . Progression to type 2 diabetes in women with a known history of gestational diabetes: systematic review and meta‐analysis. BMJ. 2020;369:m1361. doi:10.1136/bmj.m1361 32404325 PMC7218708

[6] Reece EA . The fetal and maternal consequences of gestational diabetes mellitus. J Matern Fetal Neonatal Med. 2010;23(3):199‐203. doi:10.3109/14767050903550659 20121460

[7] Rowland J , Wilson CA . The association between gestational diabetes and ASD and ADHD: a systematic review and meta‐analysis. Sci Rep. 2021;11(1):1‐16. doi:10.1038/s41598-021-84573-3 33664319 PMC7933135

[8] Dasgupta K , Ross N , Meltzer S , et al. Gestational diabetes mellitus in mothers as a diabetes predictor in fathers: a retrospective cohort analysis. Diabetes Care. 2015;38(9):e130‐e131. doi:10.2337/dc15-0855 26116719

[9] Pace R , Rahme E , Dasgupta K . Considering parents as a unit: associations of gestational diabetes and gestational hypertension with postpartum diabetes and hypertension in couples. Pregnancy Hypertens. 2019;16:32‐37. doi:10.1016/j.preghy.2019.02.004 31056157

[10] Misri S , Kostaras X , Fox D , Kostaras D . The impact of partner support in the treatment of postpartum depression. Can J Psychiatr. 2000;45(6):554‐558. doi:10.1177/070674370004500607 10986574

[11] Guo M , Parsons J , Forbes A , et al. A qualitative study exploring partner involvement in the management of gestational diabetes mellitus: the experiences of women and partners. J Clin Nurs. 2024;33(2):653‐663. doi:10.1111/JOCN.16887 37743636

[12] Tzotzis L , Hooper ME , Douglas A , et al. The needs and experiences of women with gestational diabetes mellitus from minority ethnic backgrounds in high‐income nations: a systematic integrative review. Women Birth. 2023;36(2):205‐216. doi:10.1016/j.wombi.2022.08.006 36038477

[13] Thibaut JW , Kelley HH . The Social Psychology of Groups. John Wiley; 1959.

[14] Jackson SE , Steptoe A , Wardle J . The influence of Partner's behavior on health behavior change. JAMA Intern Med. 2015;175(3):385. doi:10.1001/jamainternmed.2014.7554 25599511

[15] Brazeau AS , Meltzer SJ , Pace R , et al. Health behaviour changes in partners of women with recent gestational diabetes: a phase IIa trial. BMC Public Health. 2018;18(1):575. doi:10.1186/s12889-018-5490-x 29716559 PMC5930949

[16] DeJonckheere M , Vaughn LM . Semistructured interviewing in primary care research: a balance of relationship and rigour. Fam Med Community Health. 2019;7(2):e000057. doi:10.1136/fmch-2018-000057 32148704 PMC6910737

[17] Palinkas LA , Horwitz SM , Green CA , Wisdom JP , Duan N , Hoagwood K . Purposeful sampling for qualitative data collection and analysis in mixed method implementation research. Adm Policy Ment Health Ment Health Serv Res. 2015;42(5):533‐544. doi:10.1007/s10488-013-0528-y PMC4012002 24193818

[18] Braun V , Clarke V . Reflecting on reflexive thematic analysis. Qual Res Sport, Exerc Health. 2019;11(4):589‐597. doi:10.1080/2159676X.2019.1628806

[19] Roberts JM . Critical realism, dialectics, and qualitative research methods. J Theory Soc Behav. 2014;44(1):1‐23. doi:10.1111/JTSB.12056

[20] Craig L , Sims R , Glasziou P , Thomas R . Women's experiences of a diagnosis of gestational diabetes mellitus: a systematic review. BMC Pregnancy Childbirth. 2020;20(1):76. doi:10.1186/s12884-020-2745-1 32028931 PMC7006162

[21] Kane JC , Elafros MA , Murray SM , et al. A scoping review of health‐related stigma outcomes for high‐burden diseases in low‐ and middle‐income countries. BMC Med. 2019;17(1):17. doi:10.1186/S12916-019-1250-8 30764819 PMC6376728

[22] Stangl AL , Earnshaw VA , Logie CH , et al. The health stigma and discrimination framework: a global, crosscutting framework to inform research, intervention development, and policy on health‐related stigmas. BMC Med. 2019;17(1):31. doi:10.1186/S12916-019-1271-3 30764826 PMC6376797

[23] Benton M , Hotung N , Bird J , Ismail K , Silverio SA . The (un)controlled body: a grounded theory analysis to conceptualise stigma for women with gestational diabetes mellitus. J Health Psychol. Published online April 16, 2024. doi:10.1177/13591053241241863 PMC11977814 38628073

[24] Sandsæter HL , Horn J , Rich‐Edwards JW , Haugdahl HS . Preeclampsia, gestational diabetes and later risk of cardiovascular disease: Women's experiences and motivation for lifestyle changes explored in focus group interviews. BMC Pregnancy Childbirth. 2019;19(1):448. doi:10.1186/s12884-019-2591-1 31775681 PMC6882194

[25] O'Connor H , Madge C . My mum's thirty years out of date. Community Work Fam. 2004;7(3):351‐369. doi:10.1080/1366880042000295754

[26] Prinjha S , Field K , Rowan K . What patients think about ICU follow‐up services: a qualitative study. Crit Care. 2009;13(2):R46. doi:10.1186/cc7769 19338653 PMC2689490

[27] Nielsen KK , Kapur A , Damm P , de Courten M , Bygbjerg IC . From screening to postpartum follow‐up – the determinants and barriers for gestational diabetes mellitus (GDM) services, a systematic review. BMC Pregnancy Childbirth. 2014;14(1):41. doi:10.1186/1471-2393-14-41 24450389 PMC3901889

[28] Ryan J , Lopian L , Le B , et al. It's not raining men: a mixed‐methods study investigating methods of improving male recruitment to health behaviour research. BMC Public Health. 2019;19(1):814. doi:10.1186/s12889-019-7087-4 31234825 PMC6591998

[29] Yuen L , Wong V . Gestational diabetes mellitus: challenges for different ethnic groups. World J Diabetes. 2015;6(8):1024‐1032. doi:10.4239/wjd.v6.i8.1024 26240699 PMC4515442

[30] McLean N , Griffin S , Toney K , Hardeman W . Family involvement in weight control, weight maintenance and weight‐loss interventions: a systematic review of randomised trials. Int J Obes. 2003;27(9):987‐1005. doi:10.1038/sj.ijo.0802383 12917703

